# CD147-Cyclophilin a Interactions Promote Proliferation and Survival of Cutaneous T-Cell Lymphoma

**DOI:** 10.3390/ijms22157889

**Published:** 2021-07-23

**Authors:** Minami Sakamoto, Tomomitsu Miyagaki, Hiroaki Kamijo, Tomonori Oka, Hikari Boki, Naomi Takahashi-Shishido, Hiraku Suga, Makoto Sugaya, Shinichi Sato

**Affiliations:** 1 Department of Dermatology, University of Tokyo Graduate School of Medicine, Tokyo 113-8655, Japan; minami19860707@gmail.com (M.S.); theblankbluesky@gmail.com (H.K.); tomonorioka16@gmail.com (T.O.); pika.the.sneaker@gmail.com (H.B.); dermatology525@gmail.com (N.T.-S.); hiraku_s2002@yahoo.co.jp (H.S.); sugayamder@gmail.com (M.S.); satos-der@h.u-tokyo.a.jp (S.S.); 2 Department of Dermatology, St. Marianna University School of Medicine, Kanagawa 216-8511, Japan; 3 Department of Dermatology, International University of Health and Welfare, Chiba 286-8520, Japan

**Keywords:** mycosis fungoides, Sézary syndrome, cutaneous T-cell lymphoma, CD147, cyclophilin A

## Abstract

CD147, a transmembrane glycoprotein that belongs to the immunoglobulin superfamily, and cyclophilin A (CypA), one of the binding partners of CD147, are overexpressed in tumor cells and associated with the progression of several malignancies, including both solid and hematological malignancies. However, CD147 and CypA involvement in cutaneous T-cell lymphoma (CTCL) has not been reported. In this study, we examined CD147 and CypA expression and function using clinical samples of mycosis fungoides (MF) and Sézary syndrome (SS) and CTCL cell lines. CD147 and CypA were overexpressed by tumor cells of MF/SS, and CypA was also expressed by epidermal keratinocytes in MF/SS lesional skin. Serum CypA levels were increased and correlated with disease severity markers in MF/SS patients. Anti-CD147 antibody and/or anti-CypA antibody suppressed the proliferation of CTCL cell lines, both in vitro and in vivo, via downregulation of phosphorylated extracellular-regulated kinase 1/2 and Akt. These results suggest that CD147-CypA interactions can contribute to the proliferation of MF/SS tumor cells in both a autocrine and paracrine manner, and that the disruption of CD147-CypA interactions could be a new therapeutic strategy for the treatment of MF/SS.

## 1. Introduction

Mycosis fungoides (MF) is the most common type of cutaneous T-cell lymphoma (CTCL) and typically presents in the form of skin patches and/or plaques, which can occasionally progress to skin tumors, with subsequent lymph node and rarely visceral organ involvement [1,2]. Sézary syndrome (SS) is the representative aggressive CTCL and is characterized by the triad of generalized erythroderma, lymphadenopathy, and leukemic involvement [1,2]. MF and SS share common features, such as epidermotropism of tumor cells and CD4, CD45RO, and CCR4 expression in tumor cells [1,3]. Patients with advanced MF and SS have poor prognosis, with 5-year survival rates of 52% and median overall survival of 63 months [4]. Although a variety of systemic therapies are currently available, there are no curative options for such patients, except for stem cell transplantation, and thus the treatment of advanced MF and SS still remains challenging [2,5,6]. Like other hematopoietic malignancies, cell to cell interactions between tumor cells through surface molecules and soluble factors contribute to survival and regulate malignant growth in the form of autocrine or paracrine loops in CTCL [7,8,9,10,11].

CD147, also called Basigin or EMMPRIN, is a transmembrane glycoprotein that belongs to the immunoglobulin superfamily, which was originally cloned from embryonal carcinoma cells and initially characterized as a regulator of matrix metalloproteinases (MMPs) [12,13]. Overexpression of CD147 has been reported in the tumor cells of a variety of solid cancers and is believed to contribute to tumor progression, invasion, and metastasis by various mechanisms, such as facilitating secretion of MMPs, driving angiogenesis, developing chemoresistance, and directly promoting proliferation of tumor cells [14,15]. Actually, a recent meta-analysis, including 53 studies on the relationship between CD147 expression and outcomes in solid cancers, revealed a significant association between CD147 overexpression and adverse tumor outcomes [16]. In addition, CD147 overexpression has also been reported in several hematological malignancies, including acute myeloblastic leukemia, multiple myeloma, Hodgkin’s lymphoma, anaplastic large cell lymphoma, adult T-cell leukemia/lymphoma, and angioimmunoblastic T-cell lymphoma [17,18,19,20]. Among them, high CD147 expression is reported to be associated with a poor prognosis in patients with acute myeloblastic leukemia and multiple myeloma [17,21]. Thus, CD147 is regarded as a potential therapeutic target in a wide variety of malignancies. CD147-Cyclophilin A (CypA) interactions are also thought to be one of the best potential therapeutic targets. CypA, which was first identified as a cytosolic binding protein of the immunosuppressive drug cyclosporin A, is a peptidylprolyl cis-trans isomerase and is mainly expressed in the cytoplasm of all tissues [22,23]. Soluble CypA binds to CD147 and exerts various biological effects, including cell migration, proliferation, and differentiation, all of which can be associated with tumor progression [24]. Similarly to CD147, high CypA expression has also been reported in various tumor tissues and cell lines [24]. Moreover, the contribution of CD147-CypA interactions to tumor cell proliferation has been demonstrated in pancreatic cancer, head and neck squamous cell carcinoma, cholangiocarcinoma, glioma, and multiple myeloma [25,26,27,28,29]. However, there have been no reports on the expression and function of CD147 and CypA in CTCL. In this study, we examined the involvement of CD147-CypA interactions in the development of MF and SS using clinical samples and cell lines.

## 2. Results

### 2.1. CD147 was Overexpressed on Malignant T Cells in MF/SS

**Figure 1:**
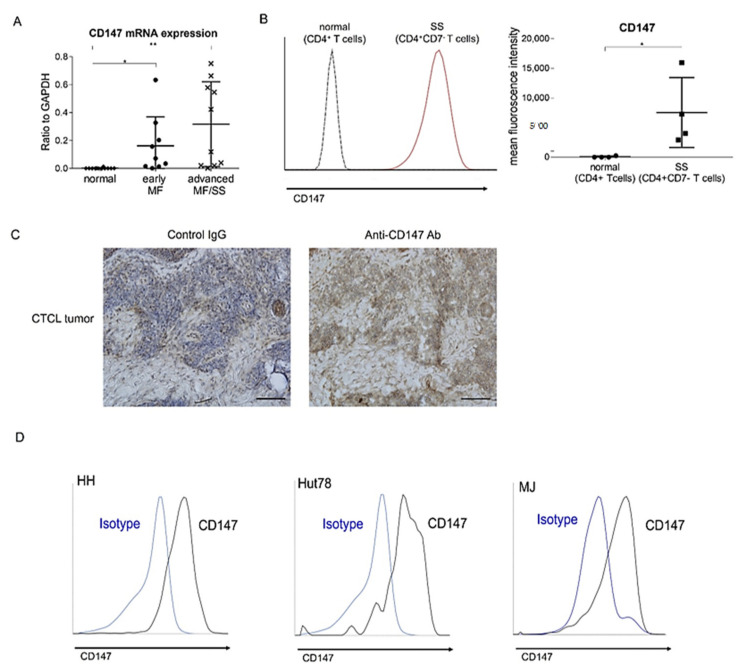
CD147 is overexpressed on tumor cells of mycosis fungoides (MF) and Sézary syndrome (SS). (**A**) Quantitative RT-PCR was performed to measure expression levels of CD147 using mRNA extracted from lesional skin of early MF (*n* = 10), advanced MF/SS (*n* = 13), and healthy skin (*n* = 12). (**B**) CD147 expression was analyzed by flow cytometry in CD4 + CD7- T cells from 4 SS patients, and CD4+ T cells from 4 healthy controls. Representative plots (left) and mean fluorescence intensity (MFI) (right) are shown. The measured values from individual patients are plotted with dots. Bars represent mean ± standard deviation. * *p* < 0.05, ** *p* < 0.01. (**C**) CD147 staining in MF/SS lesional skin (*n* = 12). Representative results are shown (original magnification ×400; scale bar = 100 μm). (**D**) CD147 expression was analyzed by flow cytometry in human cutaneous T-cell lymphoma cell lines (HH, Hut78, and MJ cells).

We first examined mRNA expression levels of CD147 in MF/SS lesional skin. CD147 mRNA levels were significantly elevated in the lesional skin of both early MF and advanced MF/SS, compared with normal skin (Figure 1A). The levels in advanced MF/SS tended to be higher than those in early MF (Figure 1A). To assess CD147 expression on tumor cells of CTCL, we next investigated CD147 expression on peripheral blood mononuclear cells (PBMCs) from SS patients and normal controls by flow cytometry. CD147 expression levels in CD4^+^CD7^-^ malignant T cells from SS patients were higher than those in CD4^+^ T cells from normal controls (Figure 1B). We also immunolabeled CTCL lesional skin for CD147. Consistent with the results of flow cytometry, CD147 was expressed diffusely on infiltrating lymphocytes with atypia (Figure 1C). Consistently, three CTCL cell lines (HH cells, Hut78 cells, and MJ cells) also expressed CD147 on their surface (Figure 1D). Thus, CD147 was overexpressed on malignant T cells of MF/SS.

### 2.2. Cyclophilin A Was Expressed on Epidermal Keratinocytes and Malignant T Cells in MF/SS

**Figure 2:**
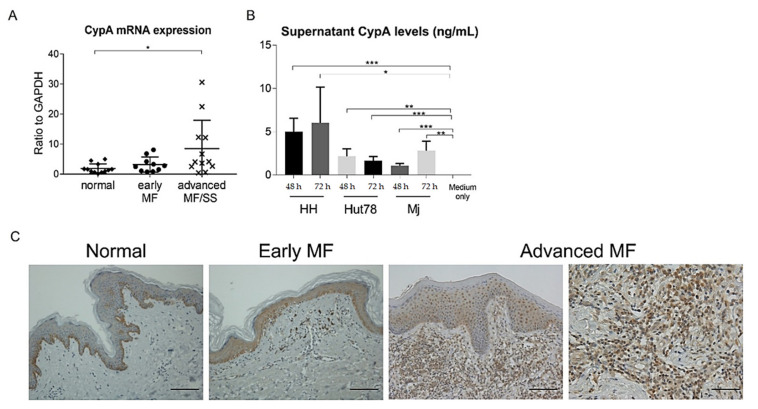
Cyclophilin A (CypA) is expressed on tumor cells and epidermal keratinocytes in mycosis fungoides (MF) and Sézary syndrome (SS). (**A**) Quantitative RT-PCR was performed to measure expression levels of CypA using mRNA extracted from lesional skin of early MF (*n* = 10), advanced MF/SS (*n* = 13), and healthy skin (*n* = 12). Bars represent mean ± 2 standard deviation. (**B**) CypA staining in MF/SS lesional skin (*n* = 7). Representative results are shown (original magnification ×200 in left three panels, and ×400 in the most right panel; scale bar = 50 μm in left three panels, and 100 μm in the right most panel. (**C**) Human cutaneous T-cell lymphoma cell lines (HH, Hut78, and MJ cells) were cultured for 24, 48, and 72 h. Supernatant CypA levels were evaluated by enzyme-linked immunosorbent assay. One representative result from two independent experiments. Data are presented as mean ± standard deviation (*n* = 4). * *p* < 0.05, ** *p* < 0.01, *** *p* < 0.005.

We next investigated CypA expression in MF/SS. CypA mRNA levels in lesional skin of advanced MF/SS, but not early MF, were higher than those in normal skin (Figure 2A). Similarly to CD147 mRNA expression levels, CypA mRNA expression levels tended to be higher in advanced MF/SS. We found that epidermal keratinocytes in MF/SS patients expressed CypA, while those in normal controls were negative for CypA, except for basal layer cells by immunohistochemistry (Figure 2B). In addition, most infiltrating lymphocytes with atypia were positive for CypA in advanced MF/SS patients (Figure 2B). We further determined CypA expression in CTCL cell lines by measuring CypA levels in the culture supernatant. CypA levels in the culture supernatant of HH cells, Hut78 cells, and MJ cells were increased compared to the control media (Figure 2C). Thus, CypA was expressed on epidermal keratinocytes in lesional skin of CTCL and malignant T cells of MF and SS.

### 2.3. Serum CypA Levels Were Increased and Correlated with Disease Severity Markers in MF/SS Patients

**Figure 3:**
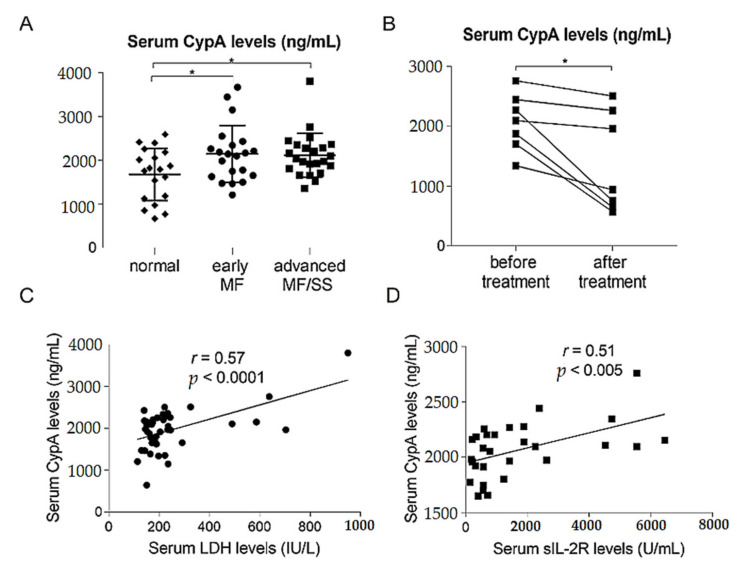
Serum cyclophilin A (CypA) levels are increased and correlated with disease severity markers in patients with mycosis fungoides (MF) and Sézary syndrome (SS). (**A**) Serum CypA levels in early MF patients (*n* = 21), advanced MF/SS patients (*n* = 23), and healthy control (*n* = 19). (**B**) Serum CypA levels in MF/SS patients (*n* = 7) before and after treatment. (**C**,**D**) Correlations between serum CypA levels and serum lactate dehydrogenase (LDH) levels (*n* = 44) (**C**) and serum soluble IL-2 receptor (sIL-2R) levels (*n* = 29) (**D**) in MF/SS patients. The measured values from individual patients are plotted with dots. Bars represent mean ± standard deviation. * *p* < 0.05.

We also measured serum CypA levels in patients with CTCL and in healthy controls. Serum CypA levels in both early MF patients and advanced MF/SS patients were significantly higher than those in healthy controls (Figure 3A). We also examined serum CypA levels before and after treatment in 7 MF/SS patients. The treatment was different for each patient, and phototherapy, local irradiation, oral etretinate, oral prednisolone, oral etoposide, mogamulizumab, gemcitabine, and a combination of these were included. Serum CypA levels significantly decreased after treatment (Figure 3B). Moreover, serum CypA levels were significantly correlated with serum lactate dehydrogenase levels and serum soluble IL-2 receptor levels, both of which are regarded as disease severity markers (Figure 3C,D). These data suggests that CypA could be involved in the development of MF/SS.

### 2.4. CD147-CypA Interactions Promote Proliferation of CTCL Cell Lines through the Phosphorylation of Extracellular Signal-Regulated Kinase 1/2 and Akt

**Figure 4:**
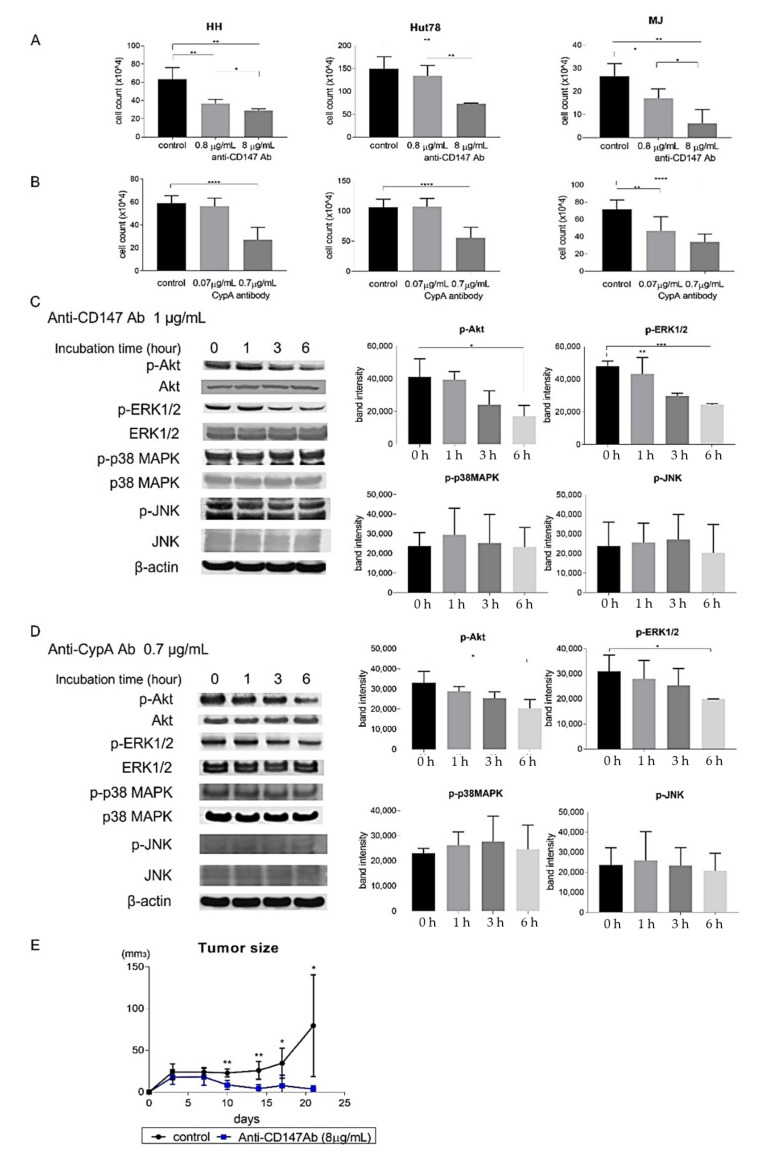
CD147-CypA interactions promoted the proliferation of cutaneous T-cell lymphoma (CTCL) cell lines. (**A**,**B**) CTCL cell lines (HH, Hut78, and MJ cells) were cultured with anti-CD147 antibody (0.8, 8 μg/mL) or anti-CypA antibody (0.07, 0.7 μg/mL) for 48 h. Viable cells were counted. Data are presented as mean ± standard deviation. (**C**,**D**) Western blotting analysis was conducted on the lysates of HH cells treated with anti-CD147 antibody (1 μg/mL) or anti-CypA antibody (0.7 μg/mL) for 0, 1, 3, or 6 h. Phosphorylation of AKT, ERK1/2, p38 MAPK, and JNK was measured. A representative picture of three independent experiments is shown. Band intensities of phosphorylated AKT, ERK1/2, p38 MAPK, and JNK at each time were calculated by Image J software. *n* = 3. Data are presented as mean ± standard deviation. (**E**) HH cells (1.0 × 10^7^) were injected into Scid-beige mice with PBS or anti-CD147 antibody (8 μg/mL). Each reagent was injected on days 0, 4, 7, 11, 14, and 18. Tumor size was calculated on days 4, 7, 11, 14, 18, and 21. Data are presented as means ± standard error of the mean (*n* = 28). * *p* < 0.05, ** *p* < 0.01, *** *p* < 0.005, **** *p* < 0.0001.

We next investigated whether CD147-CypA interactions promoted proliferation of CTCL tumor cells. Anti-CD147 antibody significantly decreased the proliferation of CTCL cell lines in a dose-dependent manner (Figure 4A). As CD147 has various binding partners [14], we also examined the effect of anti-CypA antibody. Expectedly, the antibody also significantly suppressed the proliferation of CTCL cell lines (Figure 4B). The binding of recombinant CypA to CD147 is known to activate various signaling pathways, such as extracellular signal-regulated kinase 1/2 (ERK1/2), c-jun NH2-terminal kinase (JNK), p38 mitogen-activated protein kinase (MAPK), and Akt pathways in different types of cells [24]. Consistent with those studies, Western blot analysis showed that both anti-CD147 antibody and anti-CypA antibody significantly decreased phosphorylation of ERK1/2 and AKT in HH cells (Figure 4C,D). On the other hand, expression levels of JNK and p38 MAPK were not changed (Figure 4C,D). To assess the in vivo effects of anti-CD147 antibody, we used a xenograft model [30]. Treatment with anti-CD147 antibody significantly decreased tumor formation by HH cells in SCID-beige mice in vivo (Figure 4E). These results suggest that CD147-CypA interactions promoted proliferation of CTCL cell lines, both in vitro and in vivo through the phosphorylation of ERK1/2 and Akt.

## 3. Discussion

CD147 is regarded as a therapeutic target of various malignancies. One of the most important reasons for this is that CD147 is overexpressed in malignant cells or tissues, while its expression level in normal counterparts is low [15]. Thus, targeting therapy against CD147 may have little off-target effects. In that context, a radioimmunoconjugate of metuximab, an antibody fragment targeting CD147, to the iodine-131 was developed [31]. The conjugate binds to surface CD147, resulting in internalization, and iodine-131 delivers a cytotoxic dose of gamma radiation, causing selective destruction of CD147-expressing cells. In the clinical trial for patients with hepatocellular carcinoma (HCC), the radioimmunoconjugate exhibited a significantly prolonged median time to tumor recurrence after percutaneous radiofrequency ablation, without serious adverse events [32]. Recently, the efficacy of T and NK cells transduced with a chimeric antigen receptor (CAR) that recognizes CD147 has been demonstrated in HCC using xenograft mouse models [33]. Moreover, clinical trials of CAR T-cell therapy targeting CD147 for HCC and malignant glioma are ongoing (NCT03993743, NCT04045847). In this study, we found that tumor cells of MF/SS expressed CD147 and that expression levels in tumor cells were much higher than those in CD4^+^ T cells in healthy controls. Our results suggest that CD147-targeting therapy, such as an above radioimmunoconjugate and CAR T-cell therapy might also be applied to MF/SS patients, with tolerable adverse events.

We next focused on CypA, one of the binding partners of CD147, because high expression of both CD147 and CypA has been found to be correlated with a poor prognosis in some malignancies, including esophageal carcinoma, hepatocellular carcinoma, malignant melanoma, and acute myeloid leukemia [17,34,35,36,37,38]; suggesting the importance of CD147-CypA interactions in malignancies. We found that tumor cells of MF/SS expressed CypA, as well as CD147. In addition, serum CypA levels were increased and correlated with disease severity markers in MF/SS patients. These results suggest that autocrine interactions between CD147-CypA may be involved in the development of MF/SS. In addition, we found that CypA was expressed on epidermal keratinocytes in all layers in MF/SS lesional skin, whereas CypA was mainly expressed on basal cell layers in healthy skin, which is similar to a previous report on psoriasis skin [39]. Taken together, CypA may contribute to tumor progression of MF/SS, not only in an autocrine manner, but also in a paracrine manner.

To clarify the contribution of CD147-CypA interactions to the development of MF/SS, we performed in vitro experiments using CTCL cell lines. We revealed that both anti-CD147 antibody and anti-CypA antibody diminished the proliferation of CTCL cell lines. In addition, anti-CD147 antibody suppressed tumor formation of HH cells in vivo. These results suggest that CD147-CypA interactions promote the proliferation of CTCL cell lines. Similarly, CypA induced the proliferation of CD147-expressing human pancreatic cells, human pharyngeal cell carcinoma cells, and human cholangiocarcinoma cells [25,26,27]. Downregulation of CD147 or CypA suppressed the proliferation of human glioma cells and human gastric cancer cells, which express both molecules [28,40].

CypA has the capacity to activate various signaling pathways, and the activating pathways depend on the type of cells. For example, the phosphorylation of ERK1/2 and p38 MAPK were induced in human pancreatic cells and human cholangiocarcinoma cells by CypA through CD147 [25,27]. On the other hand, anti-CypA antibody suppressed the activation of ERK1/2, JNK, and Akt pathways in human gastric cancer cells [40]. In multiple myeloma cells, anti-CD147 antibody reduced CypA-induced the phosphorylation of ERK1/2 and Akt [29]. Likewise, in CTCL cell lines, we found that both anti-CD147 antibody and anti-CypA antibody downregulated the expression of phosphorylated ERK1/2 ad Akt. Constitutive activation of both ERK1/2 and Akt signaling pathways have been reported in tumor cells of MF/SS and are thought to be involved in the progression of MF/SS [41,42,43]. Actually, phosphorylated Akt overexpression in tumor cells is significantly correlated with poor outcome [43]. Nevertheless, mutations on molecules in MAPK/ERK and Akt pathways are rare in MF/SS [44]. Thus, activation of ERK1/2 and Akt in MF/SS could be mostly induced by signaling from molecules such as CypA, but not by constitutive activation due to mutations on the pathways. In any case, ERK1/2 and Akt signaling pathways can play an important role in the proliferation of tumor cells in MF/SS.

**Figure 5:**
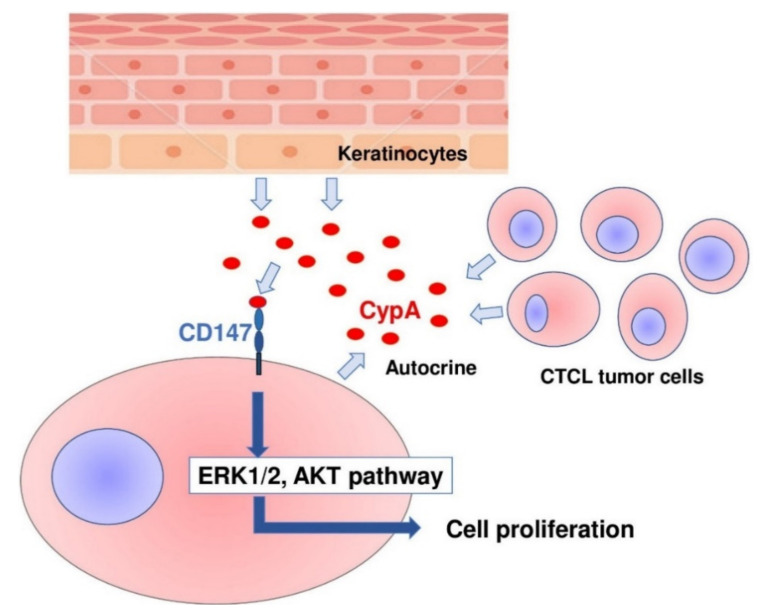
Schematic model of the main findings of this study.

In conclusion, we revealed that CD147 and CypA were overexpressed on tumor cells of MF/SS and that CypA was also expressed by epidermal keratinocytes in MF/SS lesional skin. Serum CypA levels were increased and correlated with disease severity markers in MF/SS patients. Anti-CD147 antibody and/or anti-CypA antibody suppressed the proliferation of CTCL cell lines, both in vitro and in vivo via downregulation of phosphorylated ERK1/2 and Akt. These results suggest that CD147-CypA interactions can contribute to the proliferation of MF/SS tumor cells, in both an autocrine and paracrine manner (Figure 5). The disruption of CD147-CypA interactions could be a new therapeutic strategy for the treatment of MF/SS.

## 4. Materials and Methods

### 4.1. Patients and Tissue Samples

Skin samples were collected from 23 patients with CTCL (14 male and 9 female patients; mean ± SD age: 60.2 ± 12.4 years; 19 cases of MF including 5 cases at stage IA, 5 cases at stage IB, 6 cases at stage IIB, 3 cases at stage IIIA, and 4 cases of SS, including 2 cases at stage IVA1, 2 cases at stage IVB), and 12 age-matched healthy controls. Blood samples were collected from 44 patients with CTCL (32 male and 12 female patients; mean ± SD age: 58.1 ± 16.3 years; 37 cases of MF, including 9 cases at stage IA, 9 cases at stage IB, one case at stage IIA, 5 cases at stage IIB, 13 cases at stage IIIA, and 7 cases of SS including 5 cases at stage IVA1, one case at stage IVA2, one case at stage IVB) and 20 age-matched healthy controls. PBMCs were obtained from 4 SS patients and 4 age-matched healthy controls, by density centrifugation over Ficoll-Paque (GE Healthcare, Princeton, NJ, USA). All patients with MF and SS were given diagnoses according to ISCL/EORTC criteria [1]. CTCL patients were subgrouped into early MF (IA-IIA) and advanced MF/SS (IIB-IVB), according to disease staging with TNMB classification. All samples were collected after informed consent was given during daily clinical practice. The characteristics of patients are summarized in Supplementary Table S1. The medical ethical committee of the University of Tokyo approved all described studies, and the study was conducted according to Declaration of Helsinki principles.

### 4.2. Cell Lines

HH cells (an aggressive CTCL cell line), Hut78 cells (a SS cell line), and MJ cells (a MF cell line), were a kind gift from Dr. Kazuyasu Fujii (Department of Dermatology, Kagoshima University, Kagoshima, Japan). These cell lines were authenticated by BEX Co., Ltd. (Tokyo, Japan) using a multiplex short tandem repeat assay. Hut78, HH, and MyLa cells were cultured in RPMI 1640 with 10% FBS and supplements (penicillin G sodium, streptomycin sulphate, and amphotericin B).

### 4.3. RNA Isolation and Quantitative Reverse Transcription-PCR

RNA was obtained from human skin samples with an ARNeasy Fibrous Tissue Mini Kit (Qiagen, Valencia, CA, USA). Complementary DNA was synthesized using ReverTra Ace^®^ qPCR RT Master Mix (TOYOBO, Osaka, Japan). Expression levels of CD147, CypA, and IL-4 mRNA were analyzed by a real-time PCR quantification method with THUNDERBIRD SYBR qPCR Mix (TOYOBO, Osaka, Japan) on an ABI Prism 7000 sequence detector (Applied Biosystems, Foster City, CA, USA). Glyceraldehyde-3-phosphate dehydrogenase (GAPDH) was used to normalize the mRNA. All samples were analyzed in parallel for GAPDH gene expression as an internal control. The relative expression levels of each gene were determined by the 2^−ΔΔCT^ method. Primers used were as follows: human CD147 forward, 5′-CGA GAT CCA GTG GTG GTT TG-3′ and reverse, 5′-TCG TAA GTG CCC GTG TCC-3′; human CypA forward, 5′-CAT ACG GGT CCT GGC ATC T-3′ and reverse, 5′-TGC TGG TCT TGC CAT TCC-3′; human IL-4 forward, 5′-CAC AGG CAC AAG CAG CTG AT-3′ and reverse, 5′-CTC TGG TTG GCT TCC TTC ACA-3′; human GAPDH forward, 5′-ACC CAC TCC TCC ACC TTT GA-3′ and reverse, 5′-CAT ACC AGG AAA TGA GCT TGA CAA-3′.

### 4.4. Immunohistochemistry

We also performed immunohistochemical staining for CD147 using lesional skin of MF/SS (*n* = 12). We also performed immunohistochemical staining for CypA using lesional skin of MF/SS (*n* = 7) and normal skin (*n* = 3). These sections were stained with either rabbit anti-human CD147 monoclonal antibody (Sino Biological, Beijing, China), rabbit anti-CypA polyclonal antibody (Novus Biologicals, Centennial, CO, USA), or isotype-matched control antibodies, followed by ABC staining (Vector Lab, Burlingame, CA, USA). Diaminobenzidine was used for visualizing the staining, and counterstaining with Mayer hematoxylin was performed, according to the manufactures’ instructions.

### 4.5. Flow Cytometric Analyses

We performed flow cytometric analysis using CTCL cell lines and PBMCs from SS patients, as well as healthy controls. The cells were stained with allophycocyanin-conjugated Alexa Fluor 647-conjugated anti-human CD147 antibody (Biolegend, San Diego, CA, USA), phycoerythrin (PE)-Cy7-conjugated anti-human CD4 antibody (Biolegend), and FITC-conjugated anti-human CD7 antibody (Biolegend). A FACScan flow cytometer and Cell-Quest software (BD Biosciences, San Jose, CA, USA) were used.

### 4.6. Enzyme-Linked Immunosorbent Assay

Immunoreactive CypA in sera and supernatants were quantified using a Human CypA ELISA kit (Cloud-Clone Corp, Katy, TX, USA). These assays employ the quantitative sandwich enzyme immunoassay technique. Optical densities were measured at 450 nm using a Bio-Rad Model 550 microplate reader (Bio-Rad Laboratories, Hercules, CA, USA). The concentrations were calculated from the standard curve generated by a curve-fitting program.

### 4.7. Proliferation Assays by Cell Count

Cells were plated onto 96-well plates, and anti-human CD147 antibody (Biolegend) and anti-human CypA antibody (Abcam, Cambridge, UK) were added to a final concentration as indicated. Viable cells were counted by trypan blue exclusion.

### 4.8. Western Blotting

HH cells were cultured in six-well plates in the presence or absence of anti-human CD147 antibody (1 µg/mL; Biolegend) or anti-human CypA antibody (0.7 µg/mL; Abcam) for 1, 3, or 6 h. After collecting proteins from HH cells, equal amounts of proteins were subjected to 4–12% NuPage Bis-Tris Gels (Invitrogen, Waltham, MA, USA) at 200 V for 20 min. The proteins were then transferred onto polyvinylidene fluoride membranes (Invitrogen) and blocked in 2% skim milk powder with 0.05% Tween-20 (Sigma-Aldrich, St. Louis, MO, USA) in Tris-buffered saline. The membranes were probed with Akt Antibody (Cell Signaling Technology, Danvers, MA, USA), Phospho-Akt Antibody (Cell Signaling Technology), ERK1/2 Antibody (Cell Signaling Technology), Phospho-ERK1/2 Antibody (Cell Signaling Technology), p38 MAPK Antibody (Cell Signaling Technology), Phospho-p38 MAPK Antibody (Cell Signaling Technology), SAPK/JNK Antibody (Cell Signaling Technology), Phospho-SAPK/JNK Antibody (Cell Signaling Technology), or β-actin Antibody (Cell Signaling Technology) as primary antibody overnight at 4 °C, followed by incubation in secondary antibody for 30 min at room temperature. Visualization was performed by SuperSignal West Femto Maximum Sensitivity Substrate (Thermo Scientific, Waltham, MA, USA).

### 4.9. In vivo Animal Experiments

HH cells (1.0 × 10^7^ cells) in 100 µL of RPMI were injected subcutaneously into the shaved left flank of 7–10 weeks old SCID-beige mice obtained from Charles River Laboratories (Wilmington, MA, USA). On days 0, 4, 7, 11, 14, and 18 anti-human CD147 antibody (8 µg/mL; Biolegend) was injected subcutaneously, whereas isotype IgG (R&D systems) was injected into the control group. The tumor volume was calculated on days 4, 7, 11, 14, 18, and 21 using the equation: V = volume (mm^3^) = longest diameter (mm), × shortest diameter (mm) × volume height (mm). No animals were excluded in this study. All animal experiments were approved by the Animal Research Committee of the University of Tokyo (Tokyo, Japan) on 1 February 2018 (Project code: P17-099).

### 4.10. Statistics

GraphPad Prism 7.01 software program was used for statistical analyses. Statistical analysis between the 2 groups was performed using Welch’s t test. For comparing two group values that did not follow Gaussian distribution, a two-tailed Mann–Whitney U test was used. A paired t test was used to determine significant differences in serum CypA levels before and after treatment. Correlation coefficients were determined by using Spearman’s rank correlation test. *p*-values of < 0.05 were considered statistically significant.

## Data Availability

No new data were created or analyzed in this study. Data sharing is not applicable to this article.

## Supplementary Materials

The following are available online at https://www.mdpi.com/article/10.3390/ijms22157889/s1.
