# Supplementary material for: CD147-Cyclophilin a Interactions Promote Proliferation and Survival of Cutaneous T-Cell Lymphoma
Source: Int J Mol Sci. 2021 Jul 23;22(15):7889. doi: 10.3390/ijms22157889 (PMC8346093; doi:10.3390/ijms22157889)
Supplement: Supplementary file 1 [file ijms-22-07889-s001.zip › ijms-1291647-supplementary.pdf]

Supplementary Table S1.

Case	Age	Sex	Diagnosis	Stage	RT-PCR	ELISA	IHC	Flow cytometry
**1**	82	F	Mycosis fungoides	IA (Patch)	○			
**2**	58	M	Mycosis fungoides	IA (Patch)	○			
**3**	68	F	Mycosis fungoides	IA (Patch)	○			
**4**	83	M	Mycosis fungoides	IA (Patch)	○			
**5**	62	F	Mycosis fungoides	IA (Patch)		○		
**6**	51	M	Mycosis fungoides	IA (Patch)		○		
**7**	80	M	Mycosis fungoides	IA (Patch)		○		
**8**	22	M	Mycosis fungoides	IA (Patch)		○		
**9**	39	F	Mycosis fungoides	IA (Patch)		○		
**10**	63	M	Mycosis fungoides	IA (Patch)		○		
**11**	36	F	Mycosis fungoides	IA (Patch)		○		
**12**	59	M	Mycosis fungoides	IA (Patch)			○	
**13**	50	F	Mycosis fungoides	IA (Plaque)	○			
**14**	27	F	Mycosis fungoides	IA (Plaque)		○		
**15**	61	M	Mycosis fungoides	IA (Plaque)		○	○	
**16**	54	M	Mycosis fungoides	IB (Patch)	○			
**17**	56	F	Mycosis fungoides	IB (Patch)	○			
**18**	67	F	Mycosis fungoides	IB (Patch)		○		
**19**	74	M	Mycosis fungoides	IB (Plaque)	○			
**20**	68	M	Mycosis fungoides	IB (Plaque)	○			
**21**	70	M	Mycosis fungoides	IB (Plaque)	○			
**22**	78	F	Mycosis fungoides	IB (Plaque)		○		
**23**	68	F	Mycosis fungoides	IB (Plaque)		○	○	
**24**	24	M	Mycosis fungoides	IB (Plaque)		○		
**25**	37	M	Mycosis fungoides	IB (Plaque)		○		
**26**	76	F	Mycosis fungoides	IB (Plaque)		○		
**27**	52	M	Mycosis fungoides	IB (Plaque)		○		
**28**	34	M	Mycosis fungoides	IB (Plaque)		○		
**29**	52	M	Mycosis fungoides	IB (Plaque)		○		
**30**	53	F	Mycosis fungoides	IB (Plaque)			○	
**31**	40	M	Mycosis fungoides	IIA (Plaque)		○		
**32**	75	M	Mycosis fungoides	IIB (Plaque)		○	○	
**33**	40	M	Mycosis fungoides	IIB (Tumor)	○			
**34**	63	M	Mycosis fungoides	IIB (Tumor)	○			
**35**	45	M	Mycosis fungoides	IIB (Tumor)	○	○	○	
**36**	44	M	Mycosis fungoides	IIB (Tumor)	○		○	
**37**	60	F	Mycosis fungoides	IIB (Tumor)	○			
**38**	65	F	Mycosis fungoides	IIB (Tumor)	○		○	
**39**	79	M	Mycosis fungoides	IIB (Tumor)		○		
**40**	78	M	Mycosis fungoides	IIB (Tumor)		○	○	
**41**	63	M	Mycosis fungoides	IIB (Tumor)		○		
**42**	56	M	Mycosis fungoides	IIB (Tumor)			○	
**43**	42	F	Mycosis fungoides	IIB (Tumor)			○	
**44**	64	M	Mycosis fungoides	IIB (Tumor)			○	
**45**	40	M	Mycosis fungoides	IIIA (Patch-Erythroderma)	○		○	

**46**	63	M	Mycosis fungoides	IIIA (Erythroderma)	○			
**47**	70	F	Mycosis fungoides	IIIA (Erythroderma)	○			
**48**	72	M	Mycosis fungoides	IIIA (Tumor)		○		
**49**	72	M	Mycosis fungoides	IIIA (Tumor)		○		
**50**	67	M	Mycosis fungoides	IIIA (Tumor)		○		
**51**	55	M	Mycosis fungoides	IIIA (Tumor)		○		
**52**	38	F	Mycosis fungoides	IIIA (Tumor)		○		
**53**	69	M	Mycosis fungoides	IIIA (Tumor)		○		
**54**	47	F	Mycosis fungoides	IIIA (Tumor)		○		
**55**	77	F	Mycosis fungoides	IIIA (Tumor)		○		
**56**	73	F	Mycosis fungoides	IIIA (Tumor)		○		
**57**	53	M	Mycosis fungoides	IIIA (Erythroderma)		○		
**58**	56	M	Mycosis fungoides	IIIA (Erythroderma)		○		
**59**	62	M	Mycosis fungoides	IIIA (Erythroderma)		○		
**60**	62	M	Mycosis fungoides	IIIA (Erythroderma)		○		
**61**	42	M	Sézary syndrome	IVA1	○	○	○	
**62**	64	F	Sézary syndrome	IVA1	○			
**63**	78	M	Sézary syndrome	IVA1		○	○	
**64**	74	M	Sézary syndrome	IVA1		○	○	○
**65**	69	M	Sézary syndrome	IVA1		○		
**66**	77	M	Sézary syndrome	IVA1		○		
**67**	55	M	Sézary syndrome	IVA1				○
**68**	70	M	Sézary syndrome	IVA1				○
**69**	52	M	Sézary syndrome	IVA2		○	○	
**70**	61	F	Sézary syndrome	IVA2				○
**71**	43	M	Mycosis fungoides	IVB (Tumor)			○	
**72**	58	F	Sézary syndrome	IVB	○			
**73**	67	M	Sézary syndrome	IVB	○		○	
**74**	52	M	Sézary syndrome	IVB		○		
